# PRESENCE AND PREDICTORS OF FAMILIAL AND NON-FAMILIAL AGE-INTEGRATED SOCIAL NETWORKS

**DOI:** 10.1093/geroni/igz038.615

**Published:** 2019-11-08

**Authors:** Carly Roman, Christopher Beam, Elizabeth M Zelinski

**Affiliations:** 1 USC Leonard Davis School of Gerontology, Los Angeles, California, United States; 2 USC Dornsife College of Letters, Arts and Sciences, Los Angeles, California, United States; 3 Davis School of Gerontology, University of Southern California, Los Angeles, California, United States

## Abstract

A growing older adult population living longer provides opportunities for greater age-integration, which includes reducing age-related structural barriers and increasing cross-age interactions (Riley & Riley, 2000). While research on the theoretical construct of age-integration is prevalent, empirical evidence of age-integration in older adults’ social networks is lacking (Hagestad & Uhlenburg, 2005). This study uses the National Health and Aging Trends Study to quantify and characterize age-integrated social networks in the United States, and to understand the sociodemographic predictors of these age-integrated relationships. Participants’ social networks, comprised of respondents’ spouses, household members, children, helpers, care recipients, and up to five individuals they share important things with were considered age-integrated if individuals were at least 10 years younger than the respondent. About 96% of respondents reported at least one person 10+ years younger than them. Further, these relationships were coded as familial (i.e., spouse, children, grandchildren, parents, siblings, and other relatives) and non-familial relationships (i.e., other non-relatives) and analyses predicting age-integrated relationships as a function of sociodemographic characteristics were stratified by relationship type. Weighted multilevel logistic regression analyses suggest that females have lower odds of familial and non-familial age-integration than males; compared to white and married individuals, Black and Hispanic individuals have greater odds of familial and non-familial age-integration; compared to married individuals, separated, divorced, and widowed individuals have greater odds of familial age-integration, and those who were never married have greater odds of non-familial age-integration. This foundational study reveals that sociodemographic factors differentially predict familial and non-familial age-integrated social networks.

